# Urinary Epidermal Growth Factor Level as a Noninvasive Indicator of Tubular Repair in Patients with Acute Kidney Injury

**DOI:** 10.3390/diagnostics14090947

**Published:** 2024-04-30

**Authors:** Kazutoshi Ono, Akito Maeshima, Izumi Nagayama, Taro Kubo, Takashi Yagisawa, Daisuke Nagata

**Affiliations:** 1 Division of Nephrology, Department of Internal Medicine, Jichi Medical University, Shimotsuke 329-0498, Japan; 2 Department of Nephrology and Hypertension, Saitama Medical Center, Saitama Medical University, Kawagoe 350-8550, Japan; 3 Department of Renal Surgery and Transplantation, Jichi Medical University, Shimotsuke 329-0498, Japan

**Keywords:** EGF, acute kidney injury, urinary biomarker

## Abstract

Epidermal growth factor (EGF), an essential factor for the proliferation and survival of renal tubular cells, is expressed by distal tubules and normally excreted via urine. Previous studies in rats demonstrated that acute tubular injury reduces urinary EGF levels. However, it is unclear whether urinary EGF is a suitable monitoring marker of tubular repair status after acute kidney injury (AKI) in humans. To address this question, we measured serum and urinary EGF in patients with AKI (*n* = 99) using ELISA and investigated whether urinary EGF levels were associated with the severity of tubular injury and renal prognosis. Urinary EGF was abundant in healthy controls but showed a significant decrease in AKI patients (14,522 ± 2190 pg/mL vs. 3201 ± 459.7 pg/mL, *p* < 0.05). The urinary EGF level in patients with renal AKI was notably lower than that in patients with pre-renal AKI. Furthermore, the urinary EGF level in patients with AKI stage 3 was significantly lower than that in patients with AKI stage 1. Urinary EGF levels were negatively correlated with urinary β-2MG and serum creatinine levels but positively correlated with hemoglobin levels and eGFR. Urinary EGF was not significantly correlated with urinary NAG, α-1MG, L-FABP, NGAL, KIM-1, or urinary protein concentrations. No significant correlation was observed between serum and urinary EGF levels, suggesting that urinary EGF is derived from the renal tubules rather than the blood. In living renal transplantation donors, the urinary EGF/Cr ratio was approximately half the preoperative urinary EGF/Cr ratio after unilateral nephrectomy. Collectively, these data suggest that urinary EGF is a suitable noninvasive indicator of not only the volume of functional normal renal tubules but also the status of tubular repair after AKI.

## 1. Introduction

Acute kidney injury (AKI) is a sudden reduction in kidney function leading to the accumulation of urea and other nitrogenous waste products, as well as disturbances in extracellular volume and electrolyte balances. Globally, the incidence of AKI is increasing to epidemic proportions. If not promptly diagnosed and managed, AKI is often associated with adverse outcomes and highly associated with prolonged hospitalization, increased healthcare expenditures, and elevated mortality rates, particularly in critically ill patients [1,2,3]. In clinical and epidemiological studies, the definition of AKI is based on specific criteria that have been developed sequentially over time. The Kidney Disease: Improving Global Outcomes (KDIGO) definition and staging system for AKI are the most recent and preferred [4,5]. AKI severity is classified by stages correlated with mortality risk and length of stay in the intensive care unit and hospital [6,7,8,9]. The diagnosis of AKI currently relies on serum creatinine levels and urine volume. Creatinine is a metabolite of creatine phosphate, an important source of energy for muscle movement. Therefore, serum creatinine levels vary depending on muscle mass. Due to the typical decline in muscle mass associated with aging, serum creatinine levels may not accurately assess renal function, especially in elderly individuals.

Early detection of AKI is essential to start interventions promptly, minimizing kidney damage and enhancing patient outcomes. It also allows for specific treatment strategies, such as fluid management, medication adjustments, and, in severe cases, renal replacement therapy. Urinary biomarkers play a pivotal role in assessments of AKI severity by providing a quantitative measure of renal dysfunction that aids in risk stratification. Integrating biomarkers into the diagnostic process enhances the accuracy of AKI staging and facilitates precise determination of the appropriate therapeutic approach. Biomarkers also assist in monitoring AKI progression, evaluating the efficacy of interventions, and guiding treatment adjustments based on evolving clinical statuses. This comprehensive approach enables clinicians to optimize the care of AKI patients and improve their prognosis. Ongoing research into novel biomarkers and diagnostic modalities holds promise for advancing understanding of AKI and increasing the precision of disease management strategies.

Several early diagnostic markers for AKI and markers that reflect its severity have been identified and applied clinically. Urinary neutrophil gelatinase-associated lipocalin (NGAL) [10,11,12,13], interleukin (IL)-18 [14,15], and L-type fatty acid-binding protein (L-FABP) [16,17,18] have been identified as potential markers for the early detection of kidney damage before an increase in serum creatinine levels. Kidney injury molecule 1 (KIM-1) has been suggested not only as a diagnostic biomarker but also as a marker for recovery in AKI [19,20,21,22]. A recent study proposed the combined use of two cell-cycle arrest biomarkers, insulin growth factor-binding protein 7 (IGFBP7), and tissue inhibitor of metalloproteinase 2 (TIMP-2) to predict the onset of severe AKI with significantly greater accuracy than other biomarkers [23]. However, no current biomarker provides a real-time reflection of the AKI stage or extent of recovery of tubular function.

Epidermal growth factor (EGF), initially discovered in the early 1950s [24], exhibits significant mitogenic effects in vitro across different kidney cell types and is thought to play a critical role as a mediator in post-injury renal repair [25]. Previous studies have suggested that external application of EGF affects glomerular hemodynamics, renal metabolism, tubular transport function, and eicosanoid synthesis. The EGF receptor (EGFR), which was discovered in 1986, was initially identified as a critical regulator of cell proliferation [26]. The EGFR pathway plays a pivotal role in renal organogenesis, tissue repair, and electrolyte handling. Several studies have indicated a link between dysregulation of the EGFR pathway and the onset and progression of various chronic kidney diseases, such as diabetic nephropathy, chronic allograft nephropathy, and polycystic kidney disease, promoting renal cell proliferation, fibrosis, and inflammation. Mice lacking EGFR die during or shortly after gestation due to impaired epithelial development in several organs, including the kidneys [27]. Given its role in normal development, the hypothesis that the EGFR pathway also contributes to tissue repair is plausible. Extensive research in recent decades into the role of the EGFR pathway in the kidneys has shown that EGFR signaling is significantly associated with the advancement of chronic kidney disease (CKD) [28]. Following activation of EGFRs, EGF plays diverse roles in the regulation of cell proliferation, differentiation, survival, and motility [29].

EGF expression is particularly evident in the ascending limb of Henle’s loop and the distal tubules of kidneys [30]. It has been suggested that the urinary EGF concentration reflects regulation of the signaling pathways involved in post-injury tubular recovery. Urinary EGF excretion may thus serve as a surrogate marker for the regenerative functional reserve of renal tubules [31]. However, the relationship between urinary EGF levels and disease severity, as well as the renal outcome of AKI, remains incompletely understood. To address this issue, the present study measured urinary EGF levels in AKI patients and assessed its utility as a potential biomarker reflecting the extent of recovery from tubular injury.

## 2. Materials and Methods

### 2.1. Setting and Patients

Ninety-nine patients with AKI treated at Jichi Medical University Hospital between December 2018 and February 2020 were included in this study. AKI was diagnosed and staged for severity according to the KDIGO guidelines [5]. Living-donor kidney transplantations (*n* = 16) were performed between April 2019 and March 2020 at the Jichi Medical University Hospital. Clinical data for living donors who served as controls were obtained from medical records. Written informed consent was obtained from each patient. This study was approved by the Ethical Committee on Human Research of Jichi Medical University (approval numbers A18-099, A19-057). All experiments were performed in accordance with relevant guidelines and regulations.

### 2.2. Sample and Data Collection

Urine and serum samples were collected from patients with AKI at the time of diagnosis and stored at −80 °C until analysis. Prerenal AKI was defined as recovery within 48 h and FENa < 1% or FEUN < 35%. Medical records were used to extract clinical data at the time of diagnosis regarding age, gender, urine and serum biochemical parameters, complete blood counts, complications, medications, number of days receiving renal replacement therapy (RRT), survival, and cause of death, if deceased. Serum and urine from living kidney donors who served as healthy controls (HCs; *n* = 16) were collected before nephrectomy.

### 2.3. ELISA

Urinary and serum EGF (DEG00), urinary NGAL (DLCN20), and urinary KIM-1 (DKM100) levels were measured using a Quantikine^®^ ELISA kit (R&D Systems, San Diego, CA, USA) following the manufacturer’s instructions. Urinary L-FABP levels were measured using a human FABP1/L-FABP DuoSet ELISA (DY9465-05; R&D Systems) following the manufacturer’s instructions. All standards and samples were assayed in duplicate.

### 2.4. Statistical Analysis

GraphPad Prism 8 software (GraphPad Software, San Diego, CA, USA) was used for statistical analyses. The significance of differences between means was compared using *t*-tests. When comparing the means of more than two variables, the data were analyzed using the Kruskal–Wallis test followed by the Mann–Whitney *U* test with Bonferroni correlation to adjust the probability. Correlations were determined using Spearman‘s rank correlation test coefficients. Statistical significance was defined as *p* < 0.05.

## 3. Results

### 3.1. Patient Baseline Characteristics

The baseline characteristics of the patients and HCs enrolled in this study are shown in Table 1. Seventy-one of the AKI patients were male and twenty-eight were female; the mean age of the patient group was 64.3 ± 1.5 years (mean ± SE). In comparison to HCs, AKI patients had a significantly higher frequency of diabetes and dyslipidemia. AKI patients had significantly lower serum sodium levels and significantly higher blood urea nitrogen, serum creatinine, white blood cell counts, and C-reactive protein levels. Conversely, the estimated glomerular filtration rate (eGFR), hemoglobin, and platelet counts were significantly lower in AKI patients compared with HCs. Urine tests showed significantly higher concentrations of urinary proteins, NAG, α1-MG, β2-MG, NGAL, and L-FABP in AKI patients. However, there were no significant differences between the two groups in terms of KIM-1 concentration.

### 3.2. Urinary EGF Was Significantly Decreased in AKI Patients

**Figure 1:**
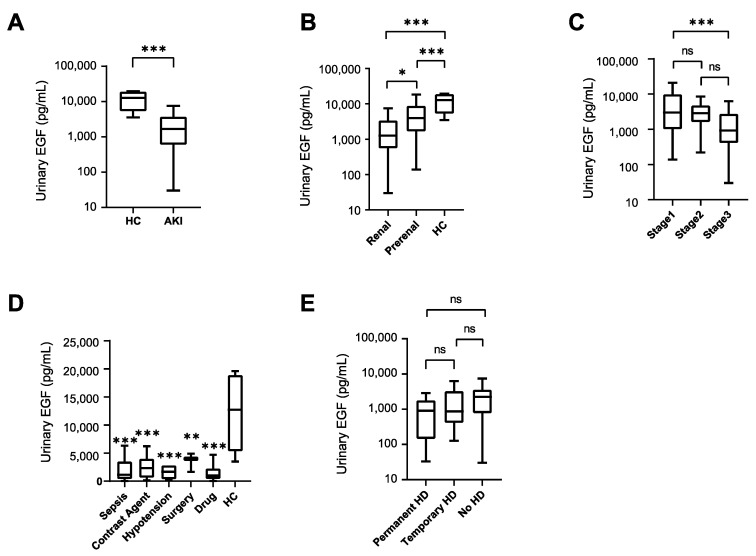
Urinary EGF levels in AKI patients. (**A**) Urinary EGF levels in the healthy control (HC) (*n* = 16) and AKI patient (*n* = 99) groups. (**B**) Urinary EGF levels in prerenal (*n* = 16) and renal AKI (*n* = 83) patients. (**C**) Urinary EGF levels and severity of AKI according to KDIGO staging criteria (stages 1–3). (**D**) Urinary EGF level by cause of AKI (sepsis 16, contrast-induced nephropathy 12, hypotension 8, drug-induced 28, miscellaneous 19). (**E**) Urinary EGF in AKI patients who required RRT and those who did not. * *p* < 0.05, ** *p* < 0.01, *** *p* < 0.001. ns, not significant. Abbreviation: KDIGO, Kidney Disease Improving Global Outcomes.

Urinary EGF levels in patients with AKI were first measured using ELISA. Urinary EGF in AKI patients was significantly lower compared with HCs (Figure 1A). Urinary EGF levels in renal AKI patients were significantly lower than in patients with prerenal AKI (Figure 1B). Urinary EGF levels in prerenal AKI patients were significantly lower than in HCs (Figure 1B). Comparing AKI patients at different stages of the disease indicated that urinary EGF was significantly lower in patients with AKI stage 3 than in patients with AKI stage 1 (Figure 1C). A significant decrease in urinary EGF was observed in renal AKI patients, regardless of the main cause of AKI (Figure 1D). There was no significant difference in urinary EGF levels between AKI patients who required RRT and those who did not (Figure 1E). 

### 3.3. Comparison of Urinary EGF with Clinical Parameters

**Figure 2:**
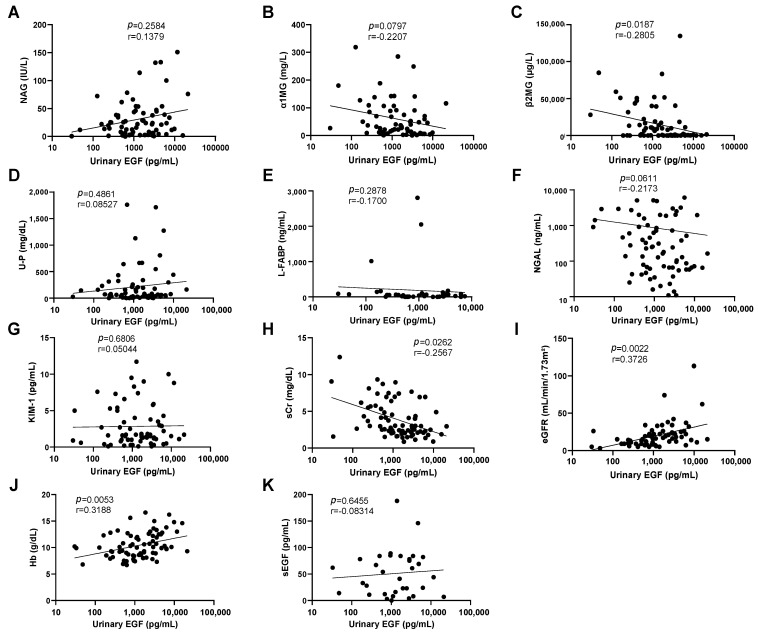
Correlation of urinary EGF with renal function and tubular damage markers. Correlations between levels of urinary EGF and NAG (**A**), α1-MG (**B**), β2-MG (**C**), urinary protein (**D**), L-FABP (**E**), NGAL (**F**), KIM-1 (**G**), serum creatinine (**H**), eGFR (**I**), hemoglobin (**J**), and serum EGF (**K**). Data from patients with renal AKI (*n* = 83) were used.

The relationship between urinary EGF levels and various clinical parameters was also investigated. No significant correlation was observed between levels of urinary EGF and urinary NAG (Figure 2A), urinary α1-MG (Figure 2B), urinary protein (Figure 2D), L-FABP (Figure 2E), urinary NGAL (Figure 2F), or urinary KIM-1 (Figure 2G). However, urinary EGF levels exhibited a negative correlation with β2-MG (Figure 2C) and serum creatinine (Figure 2H). Urinary EGF was significantly correlated with eGFR (Figure 2I) and hemoglobin (Figure 2J). Furthermore, no correlation was observed between serum EGF and urinary EGF levels (Figure 2K), indicating that urinary EGF is of renal rather than bloodstream origin. 

### 3.4. Changes in Urinary EGF Levels over Time in AKI Patients

**Figure 3:**
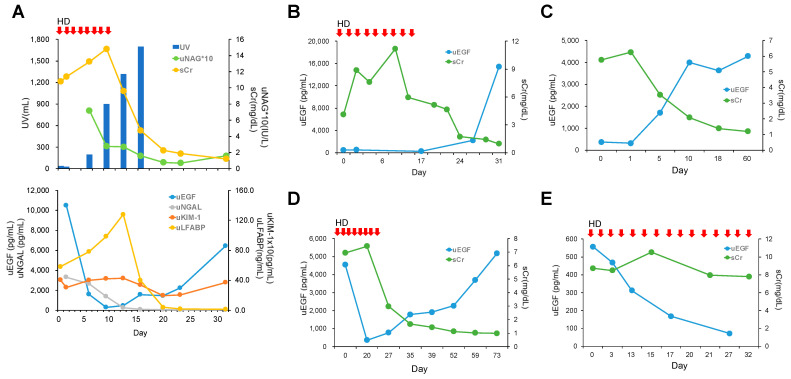
Change in urinary EGF over time in AKI patients. (**A**): Changes in serum creatinine, urinary volume, and NAG in a patient with drug-induced AKI. HD, hemodialysis. (**B**–**E**): Changes in serum creatinine and urinary EGF levels in patients with AKI of various causes, including sepsis-associated AKI (**B**), AKI after cardiac surgery (**C**), drug-induced nephropathy (**D**), and drug-induced AKI with progression to ESRD (**E**). HD, hemodialysis.

Temporal changes in urinary EGF levels were monitored alongside other AKI biomarkers in a patient with drug-induced AKI. Despite the need for RRT, the urine output gradually increased and renal function subsequently improved, such that RRT was ultimately no longer required (Figure 3A, upper graph). Upon admission, urinary EGF levels were significantly elevated but rapidly declined, concurrent with increases in other AKI biomarkers: serum creatinine, NGAL, KIM-1, and L-FABP. Urinary EGF levels returned to baseline along with the recovery of other biomarkers (Figure 3A, lower graph).

We next investigated the changes in serum creatinine and urinary EGF levels over time in patients with different causes of AKI. In cases of sepsis, urinary EGF was low and serum creatinine high, and urinary EGF increased simultaneously with a decrease in serum creatinine (Figure 3B). In cases of AKI after cardiac surgery and drug-induced AKI requiring temporary hemodialysis, urinary EGF was low and serum creatinine high, and urinary EGF increased when serum creatinine decreased (Figure 3C,D). In the case of drug-induced AKI with progression to end-stage renal disease (ESRD), the urinary EGF level continued to decrease until the patient was discharged, and the serum creatinine level remained high (Figure 3E). 

### 3.5. Urinary EGF Decreased to Half the Baseline Level after Unilateral Nephrectomy

**Figure 4:**
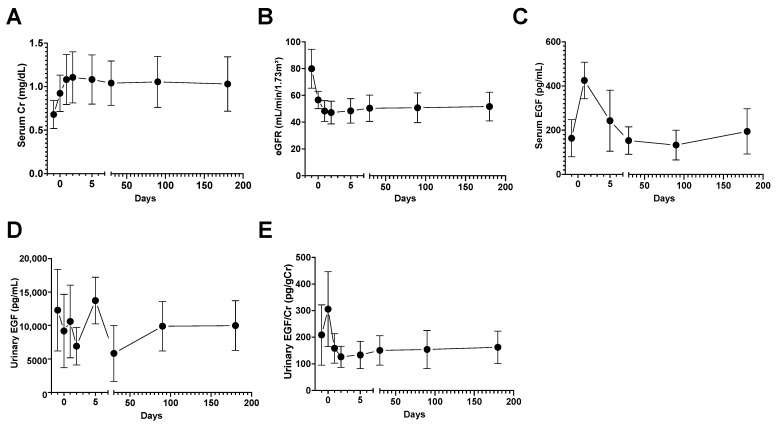
Urinary EGF in living-donor kidney transplantation patients. Serum creatinine (**A**), eGFR (**B**), serum EGF (**C**) levels, urinary EGF (**D**), and the urinary EGF/Cr ratio (**E**) in living-donor kidney transplantation patients after unilateral nephrectomy.

Finally, we measured urinary EGF levels in living kidney donors undergoing living-donor kidney transplantation. Theoretically, when one kidney is removed, the renal and tubular function and the number of normally functioning tubules in the living kidney donor should be reduced by half. Therefore, we investigated changes in urinary EGF levels in living kidney donors to determine whether the urinary EGF concentration reflects the number of normally functioning tubules. Following unilateral nephrectomy, a transient increase in serum creatinine levels was observed (Figure 4A), whereas the eGFR decreased to approximately 50% of the baseline value (Figure 4B). Conversely, serum EGF exhibited a transient increase on day 1 post-surgery but subsequently returned to preoperative levels (Figure 4C). Urinary EGF concentrations decreased after nephrectomy (Figure 4D). After nephrectomy, the urinary EGF/creatinine ratio decreased to approximately 50% of the preoperative ratio (Figure 4E). These findings suggest that the urinary EGF level accurately reflects renal function, particularly the number of functional, normal renal tubules. 

## 4. Discussion

Previous investigations reported that urinary EGF holds promise as a valuable biomarker that reflects disease activity across various kidney disorders, including lupus nephritis [32], diabetic kidney disease [33,34,35], IgA nephropathy [36,37], and autosomal dominant polycystic kidney disease [38]. Higher levels of EGF have been correlated with improved therapeutic responses and disease remission in various glomerular conditions [39,40]. Urinary EGF has emerged as a potential biomarker for predicting antibody-mediated rejection in renal transplant recipients [41]. Notably, lower urinary EGF levels have been associated with the progression of CKD and seem to mirror the extent of tubulointerstitial damage [42]. Urinary EGF has also shown promise as a useful biomarker for predicting CKD progression in the pediatric context [43]. Furthermore, urinary EGF is correlated with the eGFR slope and serves as an independent predictor of renal prognosis in individuals with CKD [44]. Notably, urinary EGF levels are significantly reduced in infants with AKI [45,46,47,48] and critically ill neonates with AKI [49].

In this study, a notable decline in urinary EGF levels was observed in AKI patients relative to HCs. Patients with renal AKI exhibited significantly lower urinary EGF levels compared to those with pre-renal AKI. Moreover, a marked reduction in urinary EGF was observed in individuals with AKI stage 3 compared to patients with AKI stage 1. Collectively, these data suggest that urinary EGF is a useful noninvasive indicator of the severity of acute tubular damage following AKI. Our investigation revealed that urinary EGF levels recover post-AKI (Figure 3). Notably, cases of AKI necessitating transient RRT exhibited a restoration of urinary EGF concentrations (Figure 3B,D). Conversely, persistently low levels of urinary EGF were observed in cases requiring permanent RRT (Figure 3E). These findings strongly suggest that urinary EGF mirrors the extent of tubular repair following AKI. The results further suggest that urinary EGF is a suitable gauge of the degree of recovery from tubular damage in cases of renal impairment. Interestingly, urinary EGF in living kidney donors decreased to nearly half of the preoperative level after kidney extraction (Figure 4), which likely reflects the number of normally functioning tubules. Hence, urinary EGF holds promise as a clinically applicable indicator of the extent of recovery from tubular damage. Building upon these results, it is conceivable that renal prognosis could be predicted by integrating the urinary EGF level with well-established AKI biomarkers. 

Downregulation of EGF expression in the kidneys post-ischemia-reperfusion injury plays a pivotal role as a mitogenic signal for regenerative responses following renal damage [50]. Studies in rats have demonstrated an immediate decrease in urinary EGF levels subsequent to ischemic events [51]. Human investigations further revealed a direct correlation between reduced urinary EGF levels and the onset of AKI, with urinary EGF concentrations aligning closely with creatinine clearance rates [52]. In the case of ischemic AKI, a notable surge in urinary EGF levels is observed at 7 days, which is not only correlated with recovery of kidney function but also associated with higher mortality rates at 3 months [53]. Recent studies involving AKI patients hospitalized with COVID-19 have shed light on the association between urinary EGF and adverse kidney outcomes, suggesting the potential for use of urinary EGF as a tool for tracking progression and recovery in kidney disease patients [54]. This study provides compelling evidence supporting a link between urinary EGF levels and AKI severity. The inclusion of urinary EGF in conjunction with standard clinical parameters holds promise for enhancing the accuracy of disease prediction across diverse CKD populations, encompassing a broad spectrum of causes and stages. Further study will be needed to clarify this issue.

Our study has several limitations. First, this study involved a small group of participants from a single center, which may not fully represent the broad spectrum of AKI. Thus, further validation involving a larger cohort of AKI patients is necessary. Because there were no consistent data regarding daily or hourly trends in urine output, AKI was defined based solely on serum creatinine criteria, raising the possibility of missing other AKI events. Additionally, in this study, urinary EGF levels at admission were not a reliable predictor of ESRD progression, diminishing their value as a predictive tool. The prognostic significance of urinary EGF in patients with less severe AKI remains uncertain, as most participants in our study had severe AKI. The mechanism by which EGF production is inhibited in the ascending limb of Henle’s loop and the distal tubule during AKI remains unclear. To elucidate the predictive significance of urinary EGF measurement in AKI, it will be essential to examine changes in EGF expression in the kidneys in AKI cases. However, renal biopsy is invasive and therefore not routinely performed in patients with AKI. Although renal biopsy is conducted in cases of AKI of unknown cause to determine the underlying issue, in many cases, the cause of AKI is already known, making histological evaluation unnecessary, as treatment decisions can be made without biopsy results. Consequently, the number of renal biopsy specimens from AKI cases is too small to conduct a quantitative analysis.

## 5. Conclusions

In conclusion, we provide supportive evidence suggesting a correlation between urinary EGF levels and the severity of AKI. Urinary EGF serves as a suitable noninvasive indicator not only for assessing the volume of functional normal renal tubules but also for evaluating the status of tubular repair following AKI.

## Figures and Tables

**Table 1:** Baseline characteristics of AKI patients and healthy controls.

	AKI		Healthy Control		
	*n* = 99		*n* = 16		*p*
Age, years, mean ± S.E.	64.3 ±	1.5	54.9 ±	2.9	0.016
BMI, kg/m^2^, mean ± S.E.	24.4 ±	0.6	23.4 ±	0.8	0.472
Male gender, *n* (%)	71	(71.7)	4	(25.0)	
Weight, kg, mean ± S.E.	63.8 ±	1.7	60.2 ±	3.2	0.398
**Complications, *n* (%)**					
Diabetes	33	(33.3)	1	(6.3)	0.036
Hypertension	69	(69.7)	7	(43.8)	0.051
Dyslipidemia	36	(36.4)	1	(6.3)	0.019
Old myocardial infarction	7	(7.1)	0	(0.0)	0.591
Old cerebral infarction	4	(4.0)	0	(0.0)	0.637
Angina pectoris	11	(11.1)	0	(0.0)	0.223
Chronic obstructive pulmonary disease	1	(1.0)	0	(0.0)	0.706
Liver disease	5	(5.1)	0	(0.0)	0.608
**Hematological data, mean ± S.E.**					
Na, mEq/L	138 ±	0.6	142.1 ±	0.5	0.003
K, mEq/L	4.4 ±	0.1	4.2 ±	0.1	0.339
Cl, mEq/L	104 ±	0.6	105.9 ±	0.6	0.111
BUN, mg/dL	53 ±	3.0	14.1 ±	0.8	<0.0001
Creatinine, mg/dL	4.02 ±	0.31	0.68 ±	0.04	<0.0001
eGFR, mL/min	20.1 ±	1.8	79.9 ±	3.6	<0.0001
Hemoglobin, mg/dL	10.4 ±	0.2	13.3 ±	0.2	<0.0001
Platelets, ×10^4^/µL	18.1 ±	1.0	26.1 ±	1.7	0.004
WBC, ×10^3^/µL	9.4 ±	0.6	5.7 ±	0.4	0.020
CRP, mg/dL	7.2 ±	0.9	0.24 ±	0.1	0.001
**Urinalysis, mean ± S.E.**					
Urinary protein, mg/dL	221.3 ±	45.1	7.8 ±	2.4	<0.0001
NAG, IU/L	31.0 ±	3.5	4.2 ±	1.2	<0.0001
α1MG, mg/L	58.1 ±	7.5	3.4 ±	0.8	<0.0001
β2MG, µg/L	12,221 ±	2370.3	162.9 ±	32.2	0.003
NGAL, ng/mL	867.3 ±	150.4	21.8 ±	8.4	<0.0001
KIM-1, ng/mL	2.8 ±	0.3	1.5 ±	0.7	0.115
L-FABP, ng/mL	161.7 ±	66.7	2.1 ±	0.6	<0.0001
FENa, %	3.7 ±	0.7	n.d.		-
FEUrea, %	35.7 ±	2.3	n.d.		-
EGF, pg/mL	3200.9 ±	456.8	12,278 ±	1528.9	<0.001

Age, sex, complications, serum sodium, potassium, chloride, creatinine, BUN, eGFR, hemoglobin, WBC, platelets, urinary protein, NAG, α1-MG, β2-MG, NGAL, KIM-1, L-FABP, FENa, and FEUN in AKI patients (*n* = 99) (sepsis 16, contrast-induced nephropathy 12, hypotension 8, drug-induced 28, miscellaneous 19) and healthy controls at baseline. Data were collected from healthy controls and AKI patients at the initial visit. Abbreviations: AKI, acute kidney injury; BMI, body mass index; BUN, blood urea nitrogen; eGFR, estimated glomerular filtration rate; WBC, white blood cell; CRP, C-reactive protein; NAG, N-acetyl-β-D-glucosaminidase; α1-MG, α1 microglobulin; β2-MG, β2 microglobulin, KIM-1, kidney injury molecule-1; NGAL, neutrophil gelatinase-associated lipocalin; L-FABP, liver-type fatty acid-binding protein; FENa, fractional excretion of sodium; FEurea, fractional excretion of urea.

## Data Availability

The datasets used and analyzed in the current study are available from the corresponding author upon reasonable request.
